# Exacerbation of cardiovascular ageing by diabetes mellitus and its associations with acyl-carnitines

**DOI:** 10.18632/aging.203144

**Published:** 2021-06-04

**Authors:** Fei Gao, Jean-Paul Kovalik, Xiaodan Zhao, Vivian JM. Chow, Hannah Chew, Louis LY. Teo, Ru San Tan, Shuang Leng, See Hooi Ewe, Hong Chang Tan, Tsze Yin Tan, Lye Siang Lee, Jianhong Ching, Bryan MH. Keng, Liang Zhong, Woon-Puay Koh, Angela S. Koh

**Affiliations:** 1 National Heart Centre Singapore, Singapore; 2 Duke-NUS Medical School, Singapore; 3 Singapore General Hospital, Singapore; 4 Healthy Longevity Translational Research Programme, Yong Loo Lin School of Medicine, National University of Singapore, Singapore; 5 KK Research Centre, KK Women's and Children's Hospital, Singapore

**Keywords:** aging, diabetes, cardiovascular

## Abstract

Objective: To demonstrate differences in cardiovascular structure and function between diabetic and non-diabetic older adults. To investigate associations between acyl-carnitines and cardiovascular function as indexed by imaging measurements.

Methods: A community-based cohort of older adults without cardiovascular disease underwent current cardiovascular imaging and metabolomics acyl-carnitines profiling based on current and archived sera obtained fifteen years prior to examination.

Results: A total of 933 participants (women 56%, n=521) with a mean age 63±13 years were studied. Old diabetics compared to old non-diabetics had lower myocardial relaxation (0.8±0.2 vs 0.9±0.3, p=0.0039); lower left atrial conduit strain (12±4.3 vs 14±4.1, p=0.045), lower left atrial conduit strain rate (-1.2±0.4 vs -1.3±0.5, p=0.042) and lower ratio of left atrial conduit strain to left atrial booster strain (0.5±0.2 vs 0.7±0.3, p=0.0029). Higher levels of archived short chain acyl-carnitine were associated with present-day impairments in myocardial relaxation (C5:1; OR 1.03, p=0.011), worse left atrial conduit strain function (C5:1; OR 1.03, p=0.037). Increases in hydroxylated acyl-carnitines were associated with worse left atrial conduit strain [(C4-OH; OR 1.05, p=0.0017), (C16:2-OH; OR 1.18, p=0.037)]. Current, archived and changes in long chain acyl-carnitines were associated with cardiovascular functions [(C16; OR 1.02, p=0.002), (C20:3; OR 1.01, p=0.014), (C14:3; OR 1.12, p=0.033), (C18:1; OR 1.01, p=0.018), (C18:2; OR 1.01, p=0.028), (C20:4; OR 1.10, p=0.038)] (all p<0.05).

Conclusion: Older diabetic adults had significant impairments in left ventricular myocardial relaxation and left atrial strain, compared to older non-diabetic adults. Short chain and long chain, di-carboxyl and hydroxylated acyl-carnitines were associated with these cardiovascular functional differences.

## INTRODUCTION

Ageing is a well-known cardiovascular risk factor that heightens risks of cardiovascular disease including myocardial infarction, heart failure, and stroke [1]. However, ageing as a risk factor exists in the same milieu as other risk factors, such as obesity and diabetes mellitus. While the individual effects on the cardiovascular system by these risk factors are known, the extent, of each risk factor, superimposed onto cardiovascular ageing, is not readily appreciated. The extent to which diabetes mellitus modifies the effect of ageing on the heart, deserves deeper investigation. The clinical implication of ageing with or without diabetes may herald differences in cardiovascular phenotype, outcomes and treatments. For example, age-associated decreases in left ventricular volumes, increases in left ventricular mass index, and deteriorations in diastolic function are commonly observed in heart failure among elderly patients [2]. The fact that heart failure can occur without diabetes (or hypertension) as a main driver, implies an urgent need to differentiate the ageing phenotype, from phenotypes related to traditional risk factors [3].

The growing elderly population worldwide highlights the need for unique strategies to confront unresolved risks of heart failure burdens among the elderly [4]. The metabolome represents net profile of diverse chemicals that is influenced by genomics, transcriptomics and proteomic variability [5]. Metabolomic profiles are also influenced by environmental exposure, diet and lifestyle [6–8]. Since metabolomics provides an integrated profile, it may serve as a conglomerating tool for life course phenomena as heterogeneous as ageing. Emerging data have demonstrated the utility of metabolomics in advancing understanding of cardiovascular ageing [9–11], insulin resistance, and diabetes [12, 13]. Therefore, metabolomics may represent a novel approach that dissects new associations between cardiovascular ageing, diabetes and metabolic pathways.

Among a community cohort of participants with risk factors but free of cardiovascular disease, we illustrate structural and functional changes associated with cardiovascular ageing and qualify the impact of diabetes mellitus on cardiovascular ageing changes. In addition, by utilizing cross-sectional and archived metabolomic profiles across the participants’ lifespan, we hypothesize that metabolites may be associated with CV changes that differentiate between older adults with diabetes versus older adults without diabetes.

## MATERIALS AND METHODS

### Study population

The subjects were recruited from the Cardiac Ageing Study (CAS)^9^, a prospective study initiated in 2014 that examines characteristics and determinants of cardiovascular function in elderly adults. CAS participants were recruited from the prospective, population-based cohort, the Singapore Chinese Health Study (SCHS) [14] and directly from the local community. The current study sample consisted of men and women who participated in the baseline CAS 2014-2017 examination who had no self-reported history of physician-diagnosed cardiovascular disease (such as coronary heart disease, atrial fibrillation), stroke or cancer. We studied the subjects in three groups, comprising of young adults (age < 65 years old), and old adults (age ≥65 years), the latter old group was further categorized into diabetic and non-diabetic adults.

Written informed consent was obtained from participants upon enrolment. The SingHealth Centralised Institutional Review Board (CIRC/2014/628/C) had approved the study protocol.

### Data acquisition

All participants were examined and interviewed on one study visit by trained study coordinators. Participants completed a standardized questionnaire that included medical history and coronary risk factors. Sinus rhythm status was ascertained by resting electrocardiogram. Clinical data were obtained on the same day as assessment of echocardiography and serum collection.

Echocardiography was performed using ALOKA α10 with a 3.5 MHz probe. In each subject, standard echocardiography, which included 2-D, M-mode, pulse Doppler and tissue Doppler imaging, was performed in the standard parasternal and apical (apical 4-chamber, apical 2-chamber and apical long) views, and three cardiac cycles were recorded. E/A ratio was computed as a ratio of peak velocity flow in early diastole E (m/s) to peak velocity flow in late diastole by atrial contraction A (m/s).

Blood samples were collected on the day of echocardiography acquisition. Plasma levels of Galectin-3 (Gal-3) (ARCHITECT Galectin-3; produced by Fujirebio Diagnostics Inc for Abbott Laboratories) and B-type natriuretic peptide (BNP) (ARCHITECT BNP; produced by Fujirebio Diagnostics Inc for Abbott Laboratories) were measured on the Abbott ARCHITECT i2000SR analyzer.

Cine cardiac magnetic resonance (CMR) scans were performed using balanced fast field echo sequence (BFFE). All subjects were imaged on a 3T magnetic resonance imaging system (Ingenia, Philips Healthcare, The Netherlands) with a dStream Torso coil (maximal number of channels 32). Dedicated Qstrain software (version 2.0, Medis) was used in deriving LV and RV longitudinal strain [15]. We developed an in-house semi-automatic algorithm to track the distance (L) between the left atrioventricular junction and a user-defined point at the mid posterior LA wall on standard CMR 2- and 4-chamber views [9].

### Metabolomics profiling

Antecubital venous blood samples (20-30 ml) were taken from consenting participants in the morning; fasting was not required before blood collection. After collection, the blood samples were immediately placed on ice for transportation and were processed within 6 h to obtain serum samples, which were subsequently stored at −80° C. Additionally, archived blood samples obtained approximately 15 years prior to this assessment from subjects who had serum samples collected and stored at the time of enrolment were analyzed.

Serum metabolomic profiling analysis for acyl-carnitines was performed in the Duke-NUS Metabolomics Facility. Thawed serum samples (100 μl) were spiked with 20 μl deuterium-labelled acyl-carnitine mixture and diluted with 800 μl methanol. Extraction and measurement of acyl-carnitine were performed as previously described [16]. Data acquisition and analysis were performed on an Agilent MassHunter Workstation B.06.00 Software.

We studied serum samples obtained from our subjects during the current study period (2014-2017) (which we will refer to as *current samples*) as well as from archived samples (1999-2004) (which we will refer to as *archived samples*) collected from the study subjects at the time of their enrolment into the Singapore Chinese Health Study approximately 15 years ago. At the time of their enrolment 15 years ago, mean age of the cohort was 59±3.8 years, mean body mass index was 23±2.9 kg/metre^2^ and prevalence of diabetes mellitus, hypertension and smoking status were 6.7%, 27% and 28.3% respectively.

### Statistics

Clinical characteristics are presented as mean and standard deviation (SD) for continuous data and frequency and percentage for categorical data. We assessed the statistical significance of the differences between young and old participants as well as difference between old diabetic and old non-diabetic in old participants. Student t-test was used for continuous data and Chi-square test was used for categorical data.

We determined acyl-carnitine profiles in serum samples from the old participants, focusing on those who had complete archived and current samples (old non-diabetic: n=154; old diabetic: n=53). The list of measured metabolites is presented in Supplementary Table 1. To identify serum metabolites correlations and reduce the dimensionality of correlated metabolites, we performed sparse principal component analysis (SPCA)^9^ using a penalized matrix decomposition on data from current serum samples (Supplementary Table 2). Metabolites with >25% of values below the lower limit of quantification were excluded from analysis (C24, C26 and C28 was excluded, hence a total of 66 metabolites were analyzed in the final sample). Other missing metabolites were input with 0.01. In SPCA, we normalized the distributions of all metabolites by a logarithmic transformation. We assessed the component metabolites within the significant PCA factors, between diabetic and non-diabetic using student t-test. For those that show an association with *p*<0.05, we further performed multivariable linear regression adjusted for clinical covariates; female, BMI and 2 or more risk factors (dyslipidemia, hypertension, smoking).

To determine the association between serum metabolomic acyl-carnitine measures to CV function, univariate Cox regression was performed on *archived* metabolites and univariate logistic regression on the change in metabolite levels between current and archived levels. Further multivariate regression model was performed on metabolites that show an association with *p*<0.05 with cardiovascular (CV) function in univariate analysis adjusted for clinical covariates; female, body mass index (BMI), diabetes mellitus (DM) and 2 or more risk factors (dyslipidemia, hypertension, smoking). Two CV functions were analysed in the comparison between old DM and old non-DM groups, (1) myocardial relaxation defined as E/A<=0.9 (mean E/A 0.9 in the Old) and (2) left atrial conduit strain defined as ɛe<=13.4 (mean ɛe 13.4 in the Old).

All statistical analyses were performed using STATA 15 (College Station, Texas, USA), while the SPCA were performed by R. For all analysis, a two-tailed *P* value of <0.05 was considered significant.

## RESULTS

We studied a total of 933 participants (women 56%, n=521) with a mean age 63±13 years. Participants were classified into young (n=418) and old (n=515) groups, based on cut off age of 65 years at the time of recruitment in 2014-2017. In the old group, we further categorized them into non-diabetics (n=399) and diabetic (n=116) subgroups. Baseline clinical characteristics of the three groups is shown in Table 1.

**Table 1:** Baseline clinical characteristics of the overall cohort.

	**Young (n=418)**	**Old** **non-diabetic** **(n=399)**	**Old diabetic (n=116)**	**Total** **(n=933)**	**P-value (young vs old)**	**p-value (old diabetic vs old non-diabetic)**
**Clinical covariates**						
Age (years)	52 (10.6)	73 (4.4)	73 (4.3)	63 (12.9)	<0.0001	0.86
Female gender, n(%)	266 (63.6%)	207 (51.9%)	48 (41.4%)	521 (55.8%)	<0.0001	0.046
Body mass index (BMI) (kg/metre^2^)	24 (3.7)	23 (3.4)	24 (3.8)	24 (3.6)	0.76	0.027
Systolic blood pressure (mmHg)	127 (19.0)	146 (23.7)	145 (16.3)	137 (22.9)	<0.0001	0.56
Diastolic blood pressure (mmHg)	76 (12.6)	74 (11.1)	70 (11.0)	75 (11.9)	0.0023	0.0008
Heart rate (beats per minute)	71 (11.0)	72 (12.9)	75 (12.7)	72 (12.1)	0.034	0.080
Hypertension, n, (%)	52 (12.4%)	188 (47.1%)	94 (81.0%)	334 (35.8%)	<0.0001	<0.0001
Dyslipidemia, n, (%)	92 (22.0%)	171 (42.9%)	92 (79.3%)	355 (38.1%)	<0.0001	<0.0001
Ever smoked, n, (%)	14 (4.2%)	62 (16.1%)	35 (32.7%)	111 (13.5%)	<0.0001	<0.0001
‘CV risk factor >=2’	39 (9.3%)	129 (32.3%)	89 (76.7%)	257 (27.6%)	<0.0001	<0.0001
**Biomarkers**						
B-type natriuretic peptide (BNP) (pg/ml)	20 (27.1)	40 (37.3)	37 (37.5)	35 (36.0)	<0.0001	0.53
Galectin-3 (ng/ml)	14.4 (7.1)	16.3 (4.2)	18.3 (5.1)	16.2 (5.3)	<0.0001	0.0003
Urinary creatinine (mmol/L)	6.4 (4.5)	6.6 (5.4)	7.5 (5.0)	6.8 (5.3)	0.66	0.22
Urinary albumin (mg/L)	9.3 (6.8)	25.8 (62.6)	23.2 (30.5)	24.0 (55.2)	0.15	0.75
Urine albumin to creatinine ratio (mg/mmol)	1.9 (1.4)	4.7 (10.1)	4.0 (6.7)	4.4 (9.1)	0.14	0.56
Glycated hemoglobin (%)	5.6 (0.7)	5.9 (0.6)	6.8 (1.0)	6.0 (0.8)	0.010	<0.0001
Random glucose (mg/dL)	114 (33.7)	116 (37)	175 (71)	122 (45)	<0.0001	<0.0001

In the old group both non-diabetic and diabetic participants were similar in age (73±4.4 vs 73±4.3 years, p=0.86). The non-diabetic subgroup had more women (52% vs 41%, p=0.046) and had lower body mass index (23±3.4 vs 24±3.8 kg/m^2^, p=0.027) compared to the diabetic subgroup. Older diabetic participants had higher prevalence of hypertension (81% vs 47%, p<0.0001), dyslipidemia (79% vs 43%, p<0.0001), and smoking history (33% vs 16%, p<0.0001) compared to older non-diabetic subjects. Older diabetic participants had higher galectin levels (18.3±5.1 vs 16.3±4.2 ng/ml, p=0.0003), plasma glucose levels (175±71 vs 116±37 mg/dl, p<0.0001), plasma glycosylated haemoglobin (6.8±1.0 vs 5.9±0.6, %, p<0.0001) but similar BNP (37±37.5 vs 40±37.3, pg/ml, p=0.53), and urine microalbumin to creatinine ratio (4.0±6.7 vs 4.7±10.1, p=0.56) compared to older non-diabetics (Table 1).

Compared to the young group, participants in the overall old group had larger left ventricular wall thickness, LV mass, left atria size and volume, and lower LV function such as lower ratio of peak velocity flow in early diastole to peak velocity flow in late diastole (Supplementary Table 3).

Left ventricular and left atria sizes and structures were similar in non-diabetic and diabetic subgroups (Table 2). However, diabetic participants had lower E/A ratio (0.8±0.2 vs 0.9±0.3, p=0.0039). Lower left atrial functions were observed among diabetics compared to the non-diabetics. Diabetics had lower left atrial conduit strain (12±4.3% vs 14±4.1%, unadjusted p=0.045), lower LA conduit strain rate (-1.2±0.4 s^-1^ vs -1.3±0.5 s^-1^, unadjusted p=0.042) and lower ratio of LA conduit strain to LA booster strain (0.5±0.2 vs 0.7±0.3, adjusted p=0.0029). Pulmonary artery systolic pressure was higher among older non-diabetics, compared to older diabetics (28±7.0 vs 25±6.9 mmHg, p=0.001) (Table 2).

**Table 2:** Cardiovascular characteristics of old non-diabetic vs old diabetic.

**Echocardiography measurements**	**Old non-diabetic (n=399)**	**Diabetic (n=116)**	**Univariate p-value**	**~Adjusted P-value**
Interventricular septum thickness at end diastole (IVSD) (cm)	0.80 (0.1)	0.81 (0.2)	0.52	-
Interventricular septum thickness at end systole (IVSS) (cm)	1.3 (0.2)	1.2 (0.2)	0.76	-
Left ventricular internal diameter end diastole (LVIDD) (cm)	4.4 (0.6)	4.3 (0.6)	0.12	-
Left ventricular internal diameter end systole (LVIDS) (cm)	2.5 (0.5)	2.4 (0.5)	0.41	-
Left ventricular posterior wall end diastole (LVPWD) (cm)	0.76 (0.1)	0.77 (0.1)	0.16	-
Left ventricular posterior wall end systole (LVPWS) (cm)	1.4 (0.2)	1.5 (0.2)	0.28	-
Left ventricular outflow tract (LVOT) (cm)	2.1 (0.2)	2.0 (0.2)	0.26	-
Aortic diameter (AO) (cm)	3.0 (0.4)	3.1 (0.4)	0.084	-
Left atrium (LA) (cm)	3.6 (0.6)	3.7 (0.6)	0.55	-
Left ventricular ejection fraction (LVEF) (%)	74 (7.7)	73 (9.2)	0.11	-
Left ventricular fractional shortening (LVFS) (%)	44 (7.4)	42 (7.8)	0.12	-
Left ventricular mass (grams)	120 (49)	116 (40)	0.41	-
Left ventricular mass index (grams/m^2^)	74 (27)	70 (22)	0.14	-
Left atrial volume (ml)	35 (13)	36 (14)	0.45	-
Left atrial volume index (ml/m^2^)	21 (7.7)	22 (8.2)	0.90	-
Isovolumic relaxation time (IVRT) (ms)	103 (18)	103 (20)	0.98	-
Peak velocity flow in early diastole E (MV E peak) (m/s)	0.71 (0.2)	0.70 (0.2)	0.51	-
Peak velocity flow in late diastole by atrial contraction A (MV A peak) (m/s)	0.81 (0.2)	0.87 (0.2)	**0.005**	0.15
Ratio of MV E peak velocity: MV A peak velocity	0.91 (0.3)	0.82 (0.2)	**0.003**	**0.039**
Mitral valve flow deceleration time (MV DT) (ms)	213 (40)	222 (42)	**0.034**	0.23
Right atrial pressure (mmHg)	5.0 (1.3)	4.7 (1.7)	0.36	-
Pulmonary artery systolic pressure (PASP) (mmHg)	28 (7.0)	25 (6.9)	**0.005**	**0.001**
Peak systolic septal mitral annular velocity (Septal S′) (m/s)	0.078 (0.02)	0.077 (0.01)	0.38	**-**
Peak early diastolic septal mitral annular velocity (Septal E’) (m/s)	0.074 (0.02)	0.067 (0.02)	**0.0003**	**0.021**
Septal mitral annular velocity during atrial contraction (Septal A’) (m/s)	0.14 (0.6)	0.11 (0.02)	0.60	-
Peak systolic lateral mitral annular velocity (m/s)	0.10 (0.03)	0.10 (0.03)	0.10	-
Peak early diastolic lateral mitral annular velocity (m/s)	0.094 (0.02)	0.088 (0.02)	**0.019**	0.094
Lateral mitral annular velocity during atrial contraction (m/s)	0.12 (0.03)	0.13 (0.02)	0.51	-
Ratio of Peak velocity flow in early diastole E (MV E peak) velocity to Peak early diastolic septal mitral annular velocity (Septal E’)	10 (3.3)	11 (3.1)	**0.022**	0.34
				
**CMR measurements**	**(n=187)**	**(n=51)**		
LV global longitudinal strain (LVGLS) (%)	-21 (2.9)	-21 (2.9)	0.28	-
LV global circumferential strain (LVGCS) (%)	-22 (3.8)	-23 (3.1)	0.21	-
LV global radial strain (LVGRS) (%)	104 (25.1)	104 (19.5)	0.98	-
Right ventricular global longitudinal strain (RVGLS) (%)	-31 (5.4)	-31 (5.5)	0.84	-
LA reservoir strain (ɛs) (%)	31 (6.9)	31 (6.2)	0.98	-
LA conduit strain (ɛe) (%)	14 (4.1)	12 (4.3)	**0.045**	0.28
LA booster strain (ɛa) (%)	17 (4.7)	18 (3.9)	0.065	-
Reservoir strain rate (SRs) (1/s)	1.5 (0.5)	1.5 (0.4)	0.92	-
Conduit strain rate (SRe) (1/s)	-1.3 (0.5)	-1.2 (0.4)	**0.042**	0.30
Booster strain rate (SRa) (1/s)	-2.2 (0.7)	-2.3 (0.6)	0.19	-
Ratio of SRe/SRa	0.66 (0.3)	0.55 (0.2)	**0.006**	**0.029**
LAvolume_min_ (ml)	31 (12.6)	27 (10.1)	**0.044**	**0.016**
LAvolume_max_ (ml)	64 (18)	57 (17)	**0.017**	**0.006**
LA ejection fraction (%)	52 (8.9)	52 (7.2)	0.92	-

~adjusted for female, BMI, CV rf>2.

In the acyl-carnitine data from the current samples adjusted linear regression analyses showed that acylcarnitine Factor 4, Factor 5 and Factor 6 differentiated older participants with diabetes from non-diabetics (Table 3). Factor 4 consists of long-chain acyl-carnitines, which are intermediates of fatty acid oxidation [17]. Specifically, we observed that diabetics had lower C18:2 (58.4 vs 67.4, p=0.020), C20:4 (4.2 vs 4.9, p=0.013), C20:3 (4.3 vs 5.3, p=0.002) and C20:2 (3.9 vs 4.4, p=0.037)], compared to non-diabetics. Factor 5 and Factor 6 consists of short chain acyl-carnitines as well as di-carboxyl and hydroxylated acyl-carnitines, which are generated by alpha and omega oxidation pathways [18, 19]. The diabetics had higher C4-OH (25.1 vs 13.0, p<0.0001), C14-OH/C12-DC (8.5 vs 5.9, p<0.0001), C16-OH (7.3 vs 5.5, p=0.001) and C18-OHC/16-DC (6.2 vs 4.5, p=0.020)] compared to non-diabetics. (Table 3).

**Table 3:** Acyl-carnitine factors and significant components: comparisons between old non-diabetic vs old diabetic.

**Acyl-carnitines**	**Non-diabetic** **(n=154)**	**Diabetic** **(n=53)**	**p-value**	Adjusted Coef. (95% CI)*	Adjusted P-value*
**PCA factors**					
X1	0.05 (2.7)	-0.1 (2.7)	0.68	-	-
X2	0.06 (2.0)	-0.2 (2.6)	0.48	-	-
X3	-0.04 (2.2)	0.1 (1.8)	0.61	-	-
X4	-0.2 (2.1)	0.7 (1.9)	**0.0080**	1.0 (0.3, 1.7)	**0.008**
X5	-0.4 (2.0)	1.1 (2.6)	**<0.0001**	1.3 (0.6, 2.0)	**<0.0001**
X6	0.2 (1.1)	-0.5 (2.2)	**0.004**	-0.6 (-1.1, -0.2)	**0.009**
X7	-0.04 (1.7)	0.1 (1.9)	0.60	-	-
X8	-0.01 (1.2)	0.04 (1.3)	0.77	-	-
X9	0.08 (1.4)	-0.2 (1.2)	0.17	-	-
X10	0.03 (1.4)	-0.1 (1.4)	0.58	-	-
					
**Short chain**					
C3	543 (180)	553 (201)	0.71	-	-
C4	338 (144)	345 (174)	0.77	-	-
C4-OH	13.0 (8.0)	25.1 (16.8)	**<0.0001**	11.0 (7.5, 14.4)	**<0.0001**
C5	95.9 (36.0)	96.5 (39.8)	0.91	-	-
C5:1	15.8 (5.4)	16.5 (7.3)	0.47	-	-
					
**Medium chain**					
C10:1	85.3 (55.8)	86.0 (79.4)	0.95	-	-
C10:2	13.1 (9.4)	15.1 (11.1)	0.24	-	-
C12-OH/C10-DC	2.1 (1.1)	2.5 (1.2)	**0.012**	0.3 (-0.06, 0.7)	0.10
C8:1-OH/C6:1-DC	27.8 (14.1)	28.4 (16.2)	0.81	-	-
C8-DC	23.3 (13.4)	26.6 (13.2)	0.12	-	-
				-	-
**Long chain**					
C14:1-OH	10.6 (6.0)	11.7 (4.6)	0.22	-	-
C14:3	4.4 (2.6)	4.2 (2.5)	0.61	-	-
C14-OH/C12-DC	5.9 (3.0)	8.5 (4.6)	**<0.0001**	2.1 (1.0, 3.2)	**<0.0001**
C16	106 (26.0)	102 (29.3)	0.31	-	-
C16:1-OH/C14:1-DC	4.8 (1.9)	5.4 (2.4)	0.055	-	-
C16:2-OH	4.3 (1.8)	4.9 (2.0)	**0.040**	0.4 (-0.2, 1.0)	0.17
C16:3-OH/C14:3-DC	1.3 (0.9)	1.4 (0.9)	0.36	-	-
C16-OH	5.5 (2.7)	7.3 (3.4)	**0.0001**	1.6 (0.6, 2.5)	**0.001**
C18	38.7 (9.8)	37.3 (8.5)	0.37	-	-
C18:1	116 (33.6)	109 (28.0)	0.15	-	-
C18:1-OH/C16:1-DC	3.8 (1.9)	4.9 (2.9)	**0.0007**	0.9 (0.1, 1.6)	**0.019**
C18:2	67.4 (20.3)	58.4 (16.2)	**0.004**	-7.7 (-14.2, -1.2)	**0.020**
C18:3	5.2 (2.1)	4.6 (2.4)	0.13	-	-
C18-OH/C16-DC	4.5 (3.2)	6.2 (3.1)	**0.012**	1.3 (0.2, 2.3)	**0.020**
C20	5.3 (1.8)	5.0 (1.3)	0.36	-	-
C20:1	6.9 (2.5)	6.7 (2.2)	0.49	-	-
C20:1-OH/C18:1-DC	7.2 (4.1)	8.0 (3.5)	0.23	-	-
C20:2	4.4 (1.5)	3.9 (1.2)	**0.033**	-0.5 (-1.0, -0.03)	**0.037**
C20:2-OH/C18:2-DC	2.2 (1.3)	2.0 (1.0)	0.37	-	-
C20:3	5.3 (2.4)	4.3 (1.7)	**0.005**	-1.2 (-1.9, -0.5)	**0.002**
C20:3-OH/C18:3-DC	1.2 (0.7)	1.3 (0.8)	0.20	-	-
C20:4	4.9 (2.0)	4.2 (1.7)	**0.035**	-0.8 (-1.5, -0.2)	**0.013**
C22:1	3.1 (2.0)	2.8 (1.6)	0.54	-	-
C22:2	0.8 (1.2)	0.7 (0.4)	0.45	-	-
C22:4	1.1 (0.7)	1.1 (0.6)	0.80	-	-
C22:5	1.8 (0.8)	1.6 (0.9)	0.16	-	-

*adjusted for female, BMI, CV rf>2.

We next examined the relationship between current serum acyl-carnitine profiles and CV structure and function in older study subjects. The di-carboxyl acyl-carnitines were associated with higher risks of impairments in E/A ratio (C12-OH/C10-DC, p=0.018); C18-OH/C16-DC, p=0.038). Similarly, the di-carboxyl acyl-carnitines were also associated with worse LA conduit strain function (C12-OH/C10-DC, p=0.008); C14-OH/C12-DC, p=0.025); C16:3-OH/C14:3-DC, p=0.018). The short-chain acyl-carnitines and hydroxylated acyl-carnitines were associated with worse LA conduit strain function (C4-OH, p=0.0024); C5, p=0.024). The long chain acyl-carnitines were associated with higher risks of impairments in E/A ratio (C16, p=0.002); C18:1, p=0.046). (Table 4).

**Table 4:** Association betwe and cardiovascular function. i) Outcome: E/A<=0.9.

**Current metabolites**	**Events/total**	**OR (95% CI)**	**p-value**	Adjusted OR (95%)*	Adjusted p-value*
**Short chain**					
C3	142/207	1.0 (1.0, 1.002)	0.62	-	-
C4	142/207	1.0 (1.0, 1.001)	0.26	-	-
C4-OH	142/207	1.03 (1.001, 1.06)	**0.045**	1.02 (0.99, 1.05)	0.25
C5	142/207	1.001 (0.99, 1.009)	0.89	-	-
C5:1	139/202	0.97 (0.93, 1.02)	0.29	-	-
**Medium chain**					
C10:1	137/195	1.002 (1.0, 1.008)	0.42	-	-
C10:2	112/168	1.03 (0.99, 1.07)	0.15	-	-
C12-OH/C10-DC	142/207	1.53 (1.11, 2.10)	**0.009**	1.50 (1.07, 2.09)	**0.018**
C8:1-OH/C6:1-DC	142/207	0.99 (0.97, 1.01)	0.45	-	-
C8-DC	142/207	1.01 (0.99, 1.04)	0.29	-	-
**Long chain**					
C14:1-OH	142/207	1.03 (0.97, 1.09)	0.38	-	-
C14:3	142/207	1.06 (0.94, 1.20)	0.35	-	-
C14-OH/C12-DC	142/207	1.13 (1.02, 1.25)	**0.016**	1.11 (1.0, 1.24)	0.052
C16	142/207	1.02 (1.007, 1.03)	**0.002**	1.02 (1.008, 1.03)	**0.002**
C16:1-OH/C14:1-DC	142/207	1.16 (0.99, 1.36)	0.067	-	-
C16:2-OH	142/207	1.06 (0.90-1.25)	0.48	-	-
C16:3-OH/C14:3-DC	136/199	1.12 (0.79-1.60)	0.52	-	-
C16-OH	142/207	1.07 (0.97, 1.19)	0.19	-	-
C18	142/207	1.03 (0.99, 1.06)	0.12	-	-
C18:1	142/207	1.01 (1.0, 1.02)	**0.043**	1.01 (1.0, 1.02)	**0.046**
C18:1-OH/C16:1-DC	142/207	1.23 (1.03, 1.46)	**0.022**	1.20 (1.0, 1.43)	0.054
C18:2	142/207	1.008 (0.99, 1.02)	0.31	-	-
C18:3	139/203	1.05 (0.92, 1.21)	0.46	-	-
C18-OH/C16-DC	142/207	1.22 (1.04, 1.42)	**0.014**	1.19 (1.01, 1.41)	**0.038**
C20	142/207	1.05 (0.88, 1.25)	0.60	-	-
C20:1	142/207	0.97 (0.87, 1.10)	0.68	-	-
C20:1-OH/C18:1-DC	142/207	1.06 (0.97, 1.15)	0.19	-	-
C20:2	142/207	0.97 (0.80, 1.19)	0.80	-	-
C20:2-OH/C18:2-DC	141/206	1.04 (0.81, 1.33)	0.76	-	-
C20:3	142/207	1.09 (0.95, 1.26)	0.22	-	-
C20:3-OH/C18:3-DC	132/194	0.86 (0.58, 1.29)	0.47	-	-
C20:4	142/207	1.03 (0.89, 1.20)	0.68	-	-
C22:1	142/207	0.98 (0.84, 1.14)	0.80	-	-
C22:2	137/197	1.36 (0.72, 2.56)	0.35	-	-
C22:4	140/203	1.11 (0.69, 1.77)	0.67	-	-
C22:5	141/205	1.06 (0.74, 1.50)	0.76	-	-

*adjusted for diabetes mellitus, female, BMI, CV rf>2.

**Table:** ii) Outcome: ɛe<=13.4 %.

**Current metabolites**	**Events/total**	**OR (95% CI)**	**p-value**	Adjusted OR (95% CI) *	Adjusted p-value *
**Short chain**					
C3	87/169	1.001 (1.0, 1.002)	0.52	-	-
C4	87/169	1.001 (1.0, 1.003)	0.66	-	-
C4-OH	87/169	1.05 (1.02, 1.09)	**0.002**	1.04 (1.006, 1.08)	**0.0024**
C5	87/169	1.01 (1.002, 1.02)	**0.022**	1.01 (1.001, 1.02)	**0.024**
C5:1	85/166	1.02 (0.97, 1.08)	0.39	-	-
**Medium chain**					
C10:1	83/157	1.002 (1.0, 1.007)	0.39	-	-
C10:2	69/134	1.02 (0.98, 1.06)	0.32	-	-
C12-OH/C10-DC	87/169	1.70 (1.23, 2.33)	**0.001**	1.58 (1.13, 2.21)	**0.008**
C8:1-OH/C6:1-DC	87/169	0.99 (0.97, 1.02)	0.54	-	-
C8-DC	87/169	1.02 (1.0, 1.05)	0.062	-	-
**Long chain**					
C14:1-OH	87/169	1.09 (1.01, 1.17)	**0.019**	1.07 (1.0, 1.16)	0.051
C14:3	87/169	1.05 (0.94, 1.18)	0.41	-	-
C14-OH/C12-DC	87/169	1.17 (1.06, 1.29)	**0.002**	1.13 (1.02, 1.25)	**0.025**
C16	87/169	1.004 (0.99, 1.02)	0.42	-	-
C16:1-OH/C14:1-DC	87/169	1.09 (0.94, 1.26)	0.28	-	-
C16:2-OH	87/169	1.19 (1.005, 1.41)	**0.043**	1.15 (0.96, 1.37)	0.13
C16:3-OH/C14:3-DC	84/161	1.78 (1.16, 2.71)	**0.008**	1.70 (1.10, 2.64)	**0.018**
C16-OH	87/169	1.07 (0.96, 1.18)	0.21	-	-
C18	87/169	0.99 (0.96, 1.02)	0.60	-	-
C18:1	87/169	1.003 (0.99, 1.01)	0.49	-	-
C18:1-OH/C16:1-DC	87/169	1.13 (0.98, 1.31)	0.10	-	-
C18:2	87/169	0.99 (0.98, 1.008)	0.33	-	-
C18:3	85/167	1.05 (0.91, 1.20)	0.52	-	-
C18-OH/C16-DC	87/169	1.15 (1.008, 1.32)	**0.038**	1.08 (0.95, 1.23)	0.25
C20	87/169	1.08 (0.90, 1.28)	0.42	-	-
C20:1	87/169	1.03 (0.92, 1.17)	0.59	-	-
C20:1-OH/C18:1-DC	87/169	1.05 (0.98, 1.14)	0.19	-	-
C20:2	87/169	1.01 (0.82, 1.24)	0.91	-	-
C20:2-OH/C18:2-DC	87/168	0.95 (0.76, 1.20)	0.67	-	-
C20:3	87/169	1.02 (0.90, 1.16)	0.75	-	-
C20:3-OH/C18:3-DC	81/159	1.13 (0.75, 1.72)	0.55	-	-
C20:4	87/169	1.008 (0.87, 1.17)	0.92	-	-
C22:1	87/169	1.08 (0.92, 1.26)	0.35	-	-
C22:2	81/160	1.29 (0.76, 2.18)	0.34	-	-
C22:4	85/166	0.83 (0.52, 1.32)	0.42	-	-
C22:5	85/167	1.10 (0.77, 1.56)	0.61	-	-

*adjusted for diabetes mellitus, female, BMI, CV rf>2.

**Figure 1:**
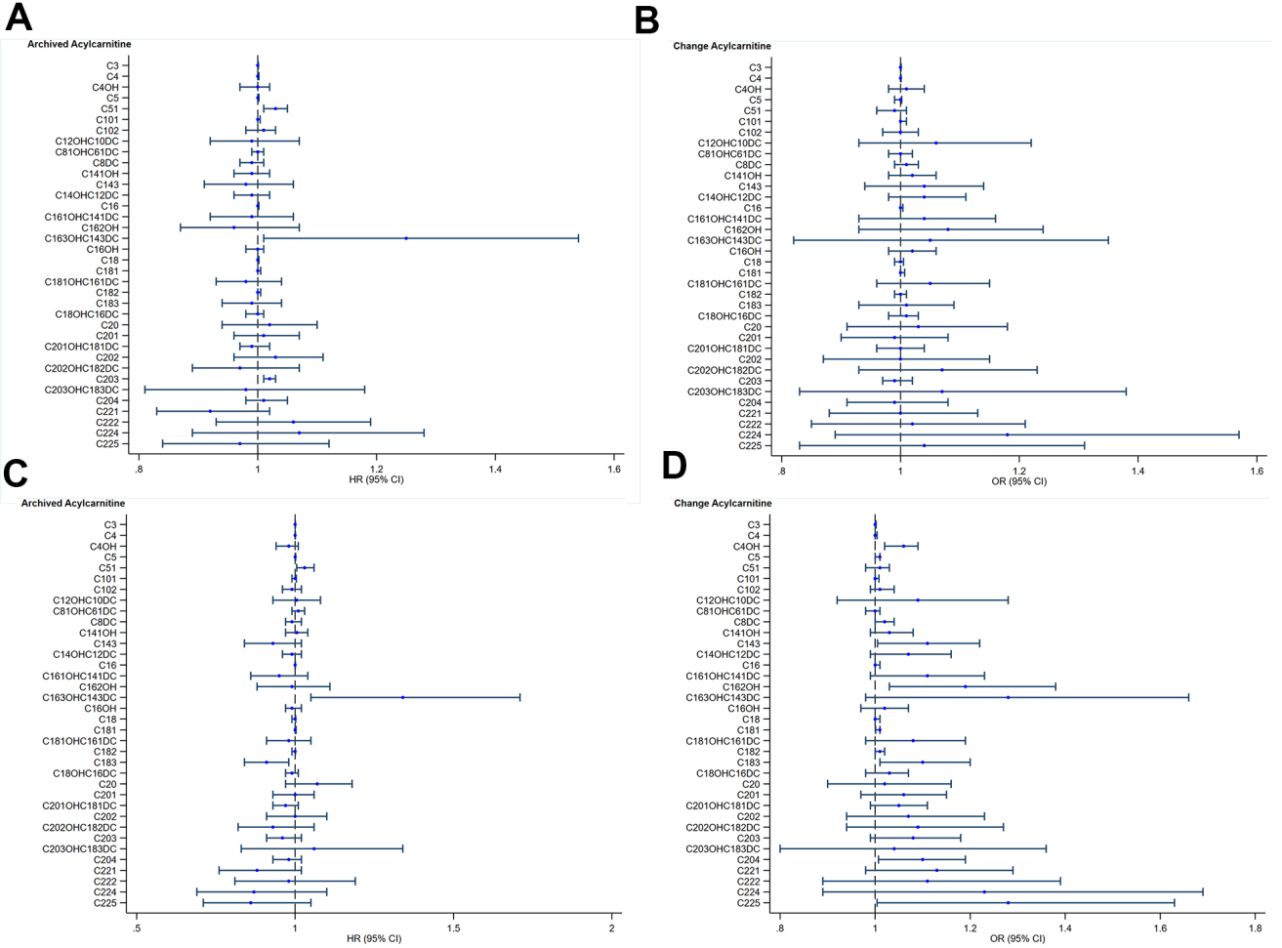
**Acyl-carnitines and cardiovascular function.** (**A**) Archived Acyl-carnitine and impaired myocardial relaxation. Blue circles and lines represent unadjusted hazard ratios (HR) for one-unit increase in archived acyl-carnitine and its 95% confidence interval (95%CI) on impaired myocardial relaxation. (**B**) Change in Acyl-carnitine and impaired myocardial relaxation. Blue circles and lines represent unadjusted odds ratios (OR) for one-unit increase in archived acyl-carnitine and its 95% confidence interval (95%CI) on impaired myocardial relaxation. (**C**) Archived Acyl-carnitine and impaired left atrial conduit strain. Blue circles and lines represent unadjusted hazard ratios (HR) for one-unit increase in archived acylcarnitine and its 95% confidence interval (95%CI) on impaired myocardial relaxation. (**D**) Change in Acyl-carnitine and impaired left atrial conduit strain. Blue circles and lines represent unadjusted odds ratios (OR) for one-unit increase in archived acyl-carnitine and its 95% confidence interval (95%CI) on impaired myocardial relaxation.

To explore associations between archived metabolites and CV function, we analyzed archived serum samples (Supplementary Table 4), as well as computed the metabolites’ change over 15-year period and assessed their hazards of association with CV function 15-years later (Supplementary Table 5). We found that higher levels of archived serum long chain acylcarnitine were associated with impairments in E/A ratio (C20:3, p=0.014) (Figure 1A). Longitudinal increases in long chain acylcarnitine over time were also associated with worse LA conduit strain function (C14:3, p=0.033); C18:1, p=0.018); C18:2, p=0.028); C18:3, p=0.019); C20:4, p=0.038); C22:5, p=0.043) (Figure 1D). Higher levels of archived short chain acylcarnitine were associated with larger hazards impairments in E/A ratio (C5:1, p=0.011) as well as with worse LA conduit strain function (C5:1, p=0.037) (Figure 1A). Higher levels of di-carboxylated acyl-carnitines were associated with worse LA conduit strain function (C16:3-OH/C14:3-DC, p=0.019) (Figure 1C). Increases in hydroxylated acyl-carnitines were also associated with worse LA conduit strain function (C4-OH, p=0.017); C16:2-OH, p=0.037) (Figure 1D).

## DISCUSSION

This was a community cohort of older adults without known prevalent cardiovascular disease. Low BNP levels in the cohort provided additional evidence that prevalent undiagnosed heart failure is probably negligible in this cohort. In determining the impact of ageing on cardiovascular structure and function in elderly adults, we recognize the difficulties of studying ageing changes in the absence of comorbidities. We used contemporary and novel imaging markers to define how the aged heart may look like in a general population setting where comorbidities invariably co-exist with human ageing. Our large sample size adjusted for these commonly occurring, age-related comorbidities.

After adjusting for comorbidity burdens, we observed definite increases in left ventricular mass index, left atrial size and left atrial volume index with ageing, along with changes in other cardiovascular *structural* markers such as left ventricular septal thickness, left ventricular internal diameter and aortic diameter. These changes have been previously reported and are thought to be a consequence of ageing [20]. In terms of function, the older adults had significant reductions in left ventricular diastolic function as noted by changes in myocardial relaxation. Observed differences in LV filling pressure did not reach clinically important thresholds, confirming that impairments in myocardial relaxation, instead of gross disturbances in diastolic function commonly observed in elderly patients with heart failure [21], typify cardiovascular ageing in this cohort.

Older adults with diabetes mellitus had similar LV *structure* to their counterparts without diabetes mellitus. In terms of cardiac function, LV and LA functional parameters revealed differences between ageing and diabetes. Older diabetic adults had significant impairments in LV myocardial relaxation, compared to older non-diabetic adults. Using advanced CMR techniques, these functional differences extended upstream to the left atrium. We observed clear differences in left atrial strain as measured by cardiac MRI. This is a novel observation which extends beyond prior reports of metabolic disturbances in left atrial strain [9], Importantly, left atrial conduit strain was the specific disturbance that differentiated diabetics and from non-diabetics, expanding on existing reports that have also observed differences in left atrial atrial strain among diabetics [22], whilst also lending support to prognostic data that has linked left atrial conduit strain to poor prognosis in cardiovascular disease cohorts [23]. This effect of diabetes on left atrial strain, exacerbated over and above effects attributable to solely ageing, should prompt greater efforts that address risks of atrial myopathy in older adults with diabetes, such as incorporating measurement of left atrial strain into clinical protocols [24] or tackling risks of left atrial myopathy in heart failure [25].

Among this cohort of older adults, the profile of acyl-carnitine metabolites differed between diabetics and non-diabetics. Diabetics had lower levels of long-chain acyl-carnitines as compared to non-diabetics. This class of metabolites is generated by mitochondrial oxidation of long-chain fatty acids. Changes in the levels of these metabolites thus reflects alterations in fatty acid oxidation, which is known to be associated with diabetes and cardiovascular disorders [26]. Older subjects with diabetes also had higher levels of dicarboxyl- and hydroxyl- carnitine metabolites. These metabolites are generally thought to be generated by alpha- and omega- fatty acid oxidation pathways [27]. Notably, these metabolites have previously been shown to be associated with increased risk of recurrent cardiovascular events as well as ischemic stroke [28]. Since patients with diabetes are known to be at higher CV risk it is perhaps understandable that older subjects with diabetes would have increased levels of these metabolites.

Interestingly, some of these acyl-carnitine metabolites were associated with cardiovascular function. Higher levels of long chain acyl-carnitines were associated with impairments in myocardial relaxation as well as with worse left atrial function. As earlier mentioned, long-chain acyl-carnitines are linked to mitochondrial fatty acid oxidation pathways. This is one of the first reports that links fuel oxidation pathways to changes in directly measured cardiac function, early disturbances in diastolic function. Previous reports have noted links between long chain acyl-carnitines and heart failure [27]. Across the clinical spectrum from heart failure with reduced ejection fraction (HFrEF), to heart failure with preserved ejection fraction (HFpEF), to non-heart failure (HF) controls, long chain acyl-carnitine levels were greater in HFrEF than HFpEF, both of which were greater than non-HF controls [29]. Our observations now directly link long chain acyl-carnitines to imaging markers of diastolic function, a pathophysiological disturbance that predominates across the clinical heart failure spectrum. In addition, levels of long chain acyl-carnitines obtained 15 years ago were associated with present-day abnormalities in these cardiovascular functions. In tandem with baseline levels of long chain acyl-carnitines from 15 years ago, interval increase in long chain acyl-carnitines predicted abnormalities in myocardial relaxation and left atrial conduit strain.

We also noted an association between increased levels of short chain, hydroxylated- and dicarboxyl- acyl-carnitines and impaired LV and atrial function, confirming these observations in baseline levels obtained 15 years ago and interval increases in these metabolites over time. These classes of fuel intermediates are likely generated by the process of alpha- and omega oxidation [18, 19]. Short chain, hydroxylated- and dicarboxyl- acyl-carnitines were specifically higher among older adults with diabetes, highlighting the importance of fuel oxidation pathways in the pathogenesis of diabetes, a connection which has been well described [26]. These pathways may also represent important treatment targets to ameliorate impact of diabetes on cardiovascular outcomes in older adults. Previous reports have noted associations between the presence of atrial fibrillation and generalized changes in metabolic pathways [30]. This is the first study to highlight alpha and omega oxidation, in association with altered left atrial function.

We acknowledge study limitations. Our observational study design does not imply causality between the metabolites and their present-day cardiovascular function. The metabolic perturbations among diabetic samples are complex, and while we could identify key pathways involved from their sera, our observational study design is unable to differentiate between adaptive versus pathogenic responses. The lower levels of certain long chain acyl-carnitines in the diabetic samples, might hypothetically represent either an adaptive response or treatment effect. In addition, metabolomics responses may differ between study groups as a consequence of dietary or lifestyle factors, which we did not account for in this analysis. A prospective longitudinal clinical trial might clarify this, alongside adjustments for medication data and duration of co-morbidities, which we did not correct for as well. However, the generally low levels of glycated haemoglobin among the groups suggest a lower risk cohort. As a community-based study focused on studying asymptomatic individuals prior to disease manifestation, our results reflect asymptomatic or preclinical phase of disease, designed to look for upstream differences that are likely subtle. Even so, true relationships between the groups, and their associations with metabolomics, may only be underestimated, and unlikely overestimated. While there may be analytic differences between non-fasting serum samples (which we used in our study) and fasting serum samples, large cohort studies face challenges in getting community elderly participants to fast for prolonged periods of time. Based on the Health Professionals Follow-up Study and Nurses’ Health Study, fasting, season of blood collection, and time of day of blood collection were not important sources of variability in measurements of most metabolites [31]. Finally, we are unable to account for residual confounding factors that may be present in such a study design.

Despite these limitations, our observations lend biological basis to previous reports that have linked fuel oxidation pathways to cardiovascular outcomes [13]*.* Our results provide basis for future work that explores the role of metabolite analysis in early detection, as a possible preventative strategy upstream in ageing.

## CONCLUSIONS

Distinct alterations in fuel oxidation pathways in short chain and long chain acyl-carnitines, di-carboxyl and hydroxylated acyl-carnitines, were associated with present-day changes in cardiovascular function. These alterations in cardiovascular function distinguished diabetic versus non-diabetic older adults. Targeting distinct fuel oxidation pathways in older adults depending on diabetes status may provide greater precision on therapeutic strategies. Investigations into acyl-carnitines early in the ageing trajectory may represent a window of opportunity to apply preventative and/or screening methods against deteriorations in cardiovascular health with ageing.

## Supplementary Material

Supplementary Tables
