# Supplementary material for: Exacerbation of cardiovascular ageing by diabetes mellitus and its associations with acyl-carnitines
Source: Aging (Albany NY). 2021 Jun 4;13(11):14785–805. doi: 10.18632/aging.203144 (PMC8221346; doi:10.18632/aging.203144)
Supplement: Supplementary Tables [file aging-13-203144-s001.pdf]

SUPPLEMENTARY TABLES

Supplementary Table 1. List of measured metabolites.

Short name	Name
C2	Acetyl carnitine
C3	Propionyl carnitine
C4	Butyryl carnitine or isobutryl carnitine
C5:1	Tiglyl carnitine or 3-methyl crotonyl carnitine
C5	Isovaleryl, 3-methylbutyryl carnitine , 2-Methylbutyryl, valeryl or pivaloyl carnitine
C4-OH	D-3-Hydroxy-butyryl carnitine, L-3-hydroxybutyryl carnitine
C6	Hexanoyl carnitine
C5-OH/C3-DC	3-Hydroxy-isovaleryl carnitine or malonyl carnitine
C4-DC/C6-OH	Methylmalonyl carnitine or succinyl carnitine
C8:1	Octenoyl carnitine
C8	Octanoyl carnitine
C5-DC	Glutaryl carnitine, ethylmalonyl carnitine
C8:1-OH/C6:1-DC	3-Hydroxy- octenoyl carnitine or hexenedioyl carnitine
C8-OH/C6-DC	3-hydroxy octanoyl carnitine or adipoyl carnitine, 3-methylglutaryl carnitine
C10:3	Decatrienoyl carnitine
C10:1	Decenoyl carnitine
C10	Decanoyl carnitine
C7-DC	Pimeloyl carnitine, heptanedioyl carnitine
C8:1-DC	Octadecenedioyl carnitine
C8-DC	Suberoyl carnitine
C12:2	-
C12:1	Dodecenoyl carnitine
C12	Lauroyl carnitine
C12:2-OH/C10:2-DC	-
C12:1-OH	Hydroxydodecenoyl carnitine
C12-OH/C10-DC	3-Hydroxy-dodecanoyl carnitine or sebacoyl carnitine
C14:3	-
C14:2	Tetradecadienoyl carnitine
C14:1	Tetradecenoyl carnitine
C14	Myristoyl carnitine
C14:3-OH/C12:3-DC	-
C14:2-OH	3-Hydroxytetradecenoylcarnitine
C14:1-OH	3-Hydroxy-tetradecenoyl carnitine
C14-OH/C12-DC	3-Hydroxy-tetradecanoyl carnitine or dodecanedioyl carnitine
C16:3	-
C16:2	Hexadecadienoyl carnitine
C16:1	Palmitoleoyl carnitine
C16	Palmitoyl carnitine
C16:3-OH/C14:3-DC	-
C16:2-OH	3-Hydroxyhexadecadienoyl carnitine
C16:1-OH/C14:1-DC	3-Hydroxy-palmitoleoyl carnitine or cis-5-tetradecenedioyl carnitine

C16-OH	3-Hydroxy-hexadecanoyl carnitine
C18:3	Linolenyl carnitine
C18:2	Linoleyl carnitine
C18:1	Oleyl carnitine
C18	Stearoyl carnitine
C18:3-OH/C16:3-DC	3-Hydroxyl-linolenyl carnitine or
C18:2-OH/C16:2-DC	3-Hydroxy-linoleyl carnitine or hexadecadienedioyl carnitine
C18:1-OH/C16:1-DC	3-Hydroxy-octadecenoyl carnitine or hexadecanedioyl carnitine
C18-OH/C16-DC	3-Hydroxy-octadecanoyl carnitine or hexadecanedioyl carnitine, thapsoyl carnitine
C20:4	Arachidonoyl carnitine
C20:3	Dihomogammalinolenyl carnitine
C20:2	-
C20:1	-
C20	Arachidoyl carnitine, eicosanoyl carnitine
C20:3-OH/C18:3-DC	-
C20:2-OH/C18:2-DC	-
C20:1-OH/C18:1-DC	Octadecenedioyl carnitine
C20-OH/C18-DC	3-Hydroxy-eicosanoyl carnitine or octadecanedioyl carnitine
C22:5	-
C22:4	-
C22:3	-
C22:2	-
C22:1	-
C22	Docosanoyl carnitine, Behenoyl carnitine

Supplementary Table 2. Factors identified by sparse principal component analysis and the associated individual components, description and variance.

Factors	Description	Components	Percentage of variance accounted
1	Medium and long-chain carnitines	C8, C121, C12, C12OHC10DC, C142, C141, C14, C163, C162, C161, C181	11
2	Short- and medium- chain dicarboxyl/ hydroxyl carnitines	C3, C4, C5, C4OH, C5OHC3DC, C4DCC6OH, C5DC, C81OHC61DC, C8OHC6DC, C102, C81DC, C8DC	6.9
3	long chain dicarboxyl/hydroxyl carnitines	C122OHC102DC, C121OH, C142OH, C141OH, C183OHC163DC, C182OHC162DC, C201, C20, C202OHC182DC, C201OHC181DC, C20OHC18DC, C221	6.8
4	Long chain carnitines	C16, C183, C182, C181, C18, C204, C203, C202, C201, C20, C202OHC182DC, C225, C224	6.4
5	Medium and long chain dicarboxyl/hydroxyl carnitines	C4, C4OH, C8DC, C12OHC10DC, C141OH, C14OHC12DC, C163OHC143DC, C162OH, C161OHC141DC, C16OH, C181OHC161DC, C18OHC16DC, C203OHC183DC, C201OHC181DC	7.7
6	Wide spectrum carnitines including odd short chain carnitines	C3, C4, C51, C5, C81OHC61DC, C102, C101, C12OHC10DC, C143, C14OHC12DC, C163OHC143DC, C161OHC141DC, C16OH, C183, C18, C181OHC161DC, C18OHC16DC, C204, C203OHC183DC, C201OHC181DC, C225, C222, C221	3.1
7	Wide spectrum carnitines including odd short chain carnitines	C2, C4OH, C6, C81, C103, C102, C101, C10, C122, C122OHC102DC, C121OH, C143, C142, C14, C141OH, C162, C161, C16, C162OH, C182, C182OHC162DC, C18OHC16DC, C202, C20, C203OHC183DC, C225, C224, C222, C22	4.3
8	Wide spectrum carnitines including odd short chain carnitines	C3, C4, C5, C4OH, C4DCC6OH, C5DC, C81OHC61DC, C8OHC6DC, C7DC, C8DC, C122, C122OHC102DC, C16OH, C183, C203OHC183DC, C202OHC182DC	2.3
9	Wide spectrum carnitines	C101, C81DC, C12OHC10DC, C141OH, C162OH, C16OH, C183, C183OHC163DC, C182OHC162DC, C18OHC16DC, C20, C203OHC183DC, C201OHC181DC, C20OHC18DC, C223, C221	2.5
10	Medium and long chain carnitines	C102, C10, C12, C121OH, C143, C14, C143OHC123DC, C163, C16, C182, C18, C204, C203, C201, C20, C221, C22	2.3

Supplementary Table 3. Cardiovascular characteristics of young vs old non-diabetic.

Echocardiography measurements	Young (n=418)	Old (n=515)	Univariate P-value	~Adjusted P-value
Interventricular septum thickness at end diastole (IVSD) (cm)	0.77 (0.1)	0.80 (0.2)	**0.001**	**0.042**
Interventricular septum thickness at end systole (IVSS) (cm)	1.2 (0.2)	1.3 (0.2)	**0.003**	**0.027**
Left ventricular internal diameter end diastole (LVIDD) (cm)	4.4 (0.4)	4.4 (0.6)	**0.019**	**0.044**
Left ventricular internal diameter end systole (LVIDS) (cm)	2.7 (0.4)	2.5 (0.5)	**<0.0001**	**<0.0001**
Left ventricular posterior wall end diastole (LVPWD) (cm)	0.7 (0.1)	0.8 (0.1)	**0.001**	**0.008**
Left ventricular posterior wall end systole (LVPWS) (cm)	1.4 (0.3)	1.4 (0.2)	0.11	-
Left ventricular outflow tract (LVOT) (cm)	2.1 (1.5)	2.1 (0.2)	0.24	-
Aortic diameter (AO) (cm)	2.8 (0.5)	3.0 (0.5)	**<0.0001**	**<0.0001**
Left atrium (LA) (cm)	3.4 (0.5)	3.7 (0.6)	**<0.0001**	**<0.0001**
Left ventricular ejection fraction (LVEF) (%)	71 (8.1)	74 (8.1)	**<0.0001**	**<0.0001**
Left ventricular fractional shortening (LVFS) (%)	40 (6.9)	43 (7.5)	**<0.0001**	**<0.0001**
Left ventricular mass (grams)	112 (32)	120 (47)	**0.004**	**0.047**
Left ventricular mass index (grams/m^2)	66 (16)	73 (26)	**<0.0001**	**<0.0001**
Left atrial volume (ml)	33 (11)	35 (13)	**0.011**	0.17
Left atrial volume index (ml/m^2)	20 (6.1)	21 (7.8)	**<0.0001**	**0.002**
Isovolumic relaxation time (IVRT) (ms)	94 (15)	103 (18)	**<0.0001**	**<0.0001**
Peak velocity flow in early diastole E (MV E peak) (m/s)	0.8 (0.2)	0.7 (0.2)	**<0.0001**	**<0.0001**
Peak velocity flow in late diastole by atrial contraction A (MV A peak) (m/s)	0.6 (0.2)	0.8 (0.2)	**<0.0001**	**<0.0001**
Ratio of MV E peak velocity: MV A peak velocity	1.4 (0.5)	0.9 (0.3)	**<0.0001**	**<0.0001**
Mitral valve flow deceleration time (MV DT) (ms)	198 (28)	215 (41)	**<0.0001**	**<0.0001**
Right atrial pressure (mmHg)	4.3 (1.3)	4.9 (1.4)	**<0.0001**	**<0.0001**
Pulmonary artery systolic pressure (PASP) (mmHg)	22 (5.4)	27 (7.0)	**<0.0001**	**<0.0001**
Peak systolic septal mitral annular velocity (Septal S′) (m/s)	0.09 (0.04)	0.08 (0.02)	**<0.0001**	**<0.0001**
Peak early diastolic septal mitral annular velocity (Septal E’) (m/s)	0.10 (0.03)	0.07 (0.02)	**<0.0001**	**<0.0001**
Septal mitral annular velocity during atrial contraction (Septal A’) (m/s)	0.1 (0.02)	0.1 (0.6)	0.50	-
Peak systolic lateral mitral annular velocity (m/s)	0.1 (0.03)	0.10 (0.03)	**<0.0001**	**<0.0001**
Peak early diastolic lateral mitral annular velocity (m/s)	0.1 (0.03)	0.09 (0.02)	**<0.0001**	**<0.0001**
Lateral mitral annular velocity during atrial contraction (m/s)	0.1 (0.03)	0.1 (0.03)	0.53	-
Ratio of Peak velocity flow in early diastole E (MV E peak) velocity to Peak early diastolic septal mitral annular velocity (Septal E’)	8.2 (2.5)	10 (3.3)	**<0.0001**	**<0.0001**
**CMR measurements**	**(n=15)**	**(n=224)**		
LV global longitudinal strain (LVGLS) (%)	-21 (3.0)	-21 (2.9)	1.00	-
LV global circumferential strain (LVGCS) (%)	-21 (4.6)	-22 (3.7)	0.36	-
LV global radial strain (LVGRS) (%)	92 (53)	104 (24)	0.10	-
Right ventricular global longitudinal strain (RVGLS) (%)	-34 (5.1)	-31 (5.4)	0.11	-
	**(n=23)**	**(n=217)**		
LA reservoir strain (εs) (%)	39 (6.2)	31 (6.8)	**<0.0001**	**<0.0001**
LA conduit strain (εe) (%)	21 (4.8)	13 (4.2)	**<0.0001**	**<0.0001**
LA booster strain (εa) (%)	17 (4.0)	17 (4.6)	0.97	-
Reservoir strain rate (SRs) (1/s)	2.0 (0.5)	1.5 (0.5)	**<0.0001**	**<0.0001**
Conduit strain rate (SRe) (1/s)	-2.5 (0.7)	-1.3 (0.5)	**<0.0001**	**<0.0001**
Booster strain rate (SRa) (1/s)	-2.4 (0.7)	-2.2 (0.7)	0.26	-
Ratio of SRe/SRa	1.1 (0.4)	0.6 (0.3)	**<0.0001**	**<0.0001**
$LAvolume_{min}$ (ml)	28 (8.8)	31 (12)	0.30	-
$LAvolume_{max}$ (ml)	65 (14)	62 (18)	0.47	-
LA ejection fraction (%)	58 (8.1)	52 (8.6)	**0.002**	**0.004**

~adjusted for female, BMI, CV rf>2.

Supplementary Table 4. Association between archived metabolites and cardiovascular function.
i) Outcome: E/A<=0.9.

Archived metabolites	Events/total	HR (95% CI)	p-value	Adjusted HR (95%)*	Adjusted p-value*
**Short chain**					
C3	124/180	1.0 (1.0, 1.001)	0.45	-	-
C4	124/180	1.0 (1.0, 1.002)	0.62	-	-
C4-OH	124/180	1.0 (0.97, 1.02)	0.59	-	-
C5	124/180	1.0 (1.0, 1.002)	0.50	-	-
C5:1	119/173	1.03 (1.01, 1.05)	**0.003**	1.03 (1.01, 1.05)	**0.011**
**Medium chain**					
C10:1	119/168	1.0 (1.0, 1.004)	0.97	-	-
C10:2	96/143	1.01 (0.98, 1.03)	0.53	-	-
C12-OH/C10-DC	124/180	0.99 (0.92, 1.07)	0.88	-	-
C8:1-OH/C6:1-DC	124/180	1.0 (0.99, 1.01)	0.81	-	-
C8-DC	124/180	0.99 (0.97, 1.01)	0.50	-	-
**Long chain**					
C14:1-OH	124/180	0.99 (0.96, 1.02)	0.47	-	-
C14:3	124/180	0.98 (0.91, 1.06)	0.69	-	-
C14-OH/C12-DC	124/180	0.99 (0.96, 1.02)	0.57	-	-
C16	124/180	1.0 (1.0, 1.002)	0.60	-	-
C16:1-OH/C14:1-DC	124/180	0.99 (0.92, 1.06)	0.74	-	-
C16:2-OH	124/180	0.96 (0.87, 1.07)	0.48	-	-
C16:3-OH/C14:3-DC	118/172	1.25 (1.01, 1.54)	**0.036**	1.19 (0.97, 1.46)	0.10
C16-OH	124/180	1.0 (0.98, 1.01)	0.61	-	-
C18	124/180	1.0 (1.0, 1.002)	0.95	-	-
C18:1	124/180	1.0 (1.0, 1.005)	0.17	-	-
C18:1-OH/C16:1-DC	124/180	0.98 (0.93, 1.04)	0.55	-	-
C18:2	124/180	1.0 (1.0, 1.005)	0.87	-	-
C18:3	121/176	0.99 (0.94, 1.04)	0.71	-	-
C18-OH/C16-DC	124/180	1.0 (0.98, 1.01)	0.88	-	-
C20	124/180	1.02 (0.94, 1.10)	0.62	-	-
C20:1	124/180	1.01 (0.96, 1.07)	0.62	-	-
C20:1-OH/C18:1-DC	124/180	0.99 (0.97, 1.02)	0.53	-	-
C20:2	124/180	1.03 (0.96, 1.11)	0.37	-	-
C20:2-OH/C18:2-DC	123/179	0.97 (0.89, 1.07)	0.55	-	-
C20:3	124/180	1.02 (1.01, 1.03)	**0.003**	1.01 (1.003, 1.03)	**0.014**
C20:3-OH/C18:3-DC	115/168	0.98 (0.81, 1.18)	0.83	-	-
C20:4	124/180	1.01 (0.98, 1.05)	0.46	-	-
C22:1	124/180	0.92 (0.83, 1.02)	0.10	-	-
C22:2	119/170	1.06 (0.93, 1.19)	0.44	-	-
C22:4	121/176	1.07 (0.89, 1.28)	0.47	-	-
C22:5	123/178	0.97 (0.84, 1.12)	0.64	-	-

*Correct for diabetes mellitus, female, BMI, CV rf>2.

ii) Outcome: εe<=13.4 %.

Archived metabolites	Events/total	HR (95% CI)	p-value	Adjusted HR (95% CI) *	Adjusted p-value *
**Short chain**					
C3	85/163	1.0 (1.0, 1.001)	0.98	-	-
C4	85/163	1.0 (1.0, 1.001)	0.42	-	-
C4-OH	85/163	0.98 (0.94, 1.01)	0.22	-	-
C5	85/163	1.0 (1.0, 1.002)	0.56	-	-
C5:1	83/159	1.03 (1.005, 1.06)	**0.018**	1.03 (1.002, 1.06)	**0.037**
**Medium chain**					
C10:1	81/151	1.0 (0.99, 1.004)	0.68	-	-
C10:2	68/130	0.99 (0.96, 1.02)	0.44	-	-
C12-OH/C10-DC	85/163	1.005 (0.93, 1.08)	0.90	-	-
C8:1-OH/C6:1-DC	85/163	1.01 (0.99, 1.03)	0.20	-	-
C8-DC	85/163	0.99 (0.97, 1.02)	0.66	-	-
**Long chain**					
C14:1-OH	85/163	1.006 (0.97, 1.04)	0.73	-	-
C14:3	85/163	0.93 (0.84, 1.02)	0.12	-	-
C14-OH/C12-DC	85/163	0.99 (0.96, 1.02)	0.66	-	-
C16	85/163	1.0 (1.0, 1.001)	0.27	-	-
C16:1-OH/C14:1-DC	85/163	0.95 (0.86, 1.04)	0.26	-	-
C16:2-OH	85/163	0.99 (0.88, 1.11)	0.83	-	-
C16:3-OH/C14:3-DC	82/155	1.34 (1.05, 1.71)	**0.017**	1.32 (1.05, 1.67)	**0.019**
C16-OH	85/163	0.99 (0.97, 1.02)	0.57	-	-
C18	85/163	1.0 (0.99, 1.002)	0.32	-	-
C18:1	85/163	1.0 (1.0, 1.003)	0.64	-	-
C18:1-OH/C16:1-DC	85/163	0.98 (0.91, 1.05)	0.51	-	-
C18:2	85/163	1.0 (0.99, 1.001)	0.12	-	-
C18:3	83/161	0.91 (0.84, 0.98)	**0.019**	0.89 (0.82, 0.96)	**0.005**
C18-OH/C16-DC	85/163	0.99 (0.97, 1.01)	0.50	-	-
C20	85/163	1.07 (0.97, 1.18)	0.16	-	-
C20:1	85/163	1.0 (0.93, 1.06)	0.89	-	-
C20:1-OH/C18:1-DC	85/163	0.97 (0.93, 1.01)	0.19	-	-
C20:2	85/163	1.0 (0.91, 1.10)	0.99	-	-
C20:2-OH/C18:2-DC	85/162	0.93 (0.82, 1.06)	0.29	-	-
C20:3	85/163	0.96 (0.91, 1.02)	0.20	-	-
C20:3-OH/C18:3-DC	79/153	1.06 (0.83, 1.34)	0.66	-	-
C20:4	85/163	0.98 (0.93, 1.02)	0.33	-	-
C22:1	85/163	0.88 (0.76, 1.02)	0.096	-	-
C22:2	79/154	0.98 (0.81, 1.19)	0.85	-	-
C22:4	83/160	0.87 (0.69, 1.10)	0.24	-	-
C22:5	83/161	0.86 (0.71, 1.05)	0.14	-	-

*adjusted for diabetes mellitus, female, BMI, CV rf>2.

Supplementary Table 5. Association between change in metabolites and cardiovascular function.
i) Outcome: εe<=13.4 %.

Changes from archived to current	Events/total	OR (95% CI)	p-value	Adjusted OR (95% CI) *	Adjusted p-value *
**Short chain**					
C3	85/163	1.0 (1.0, 1.002)	0.37	-	-
C4	85/163	1.0 (1.0, 1.004)	0.14	-	-
C4-OH	85/163	1.06 (1.02, 1.09)	**0.001**	1.05 (1.01, 1.08)	**0.017**
C5	85/163	1.01 (1.0, 1.01)	0.077	-	-
C5:1	83/159	1.01 (0.98, 1.03)	0.66	-	-
**Medium chain**					
C10:1	81/151	1.0 (1.0, 1.008)	0.25	-	-
C10:2	68/130	1.01 (0.99, 1.04)	0.32	-	-
C12-OH/C10-DC	85/163	1.09 (0.92, 1.28)	0.32	-	-
C8:1-OH/C6:1-DC	85/163	1.0 (0.98, 1.01)	0.55	-	-
C8-DC	85/163	1.02 (1.0, 1.04)	0.092	-	-
**Long chain**					
C14:1-OH	85/163	1.03 (0.99, 1.08)	0.13	-	-
C14:3	85/163	1.11 (1.005, 1.22)	**0.040**	1.12 (1.009, 1.25)	**0.033**
C14-OH/C12-DC	85/163	1.07 (0.99, 1.16)	0.076	-	-
C16	85/163	1.0 (1.0, 1.01)	0.11	-	-
C16:1-OH/C14:1-DC	85/163	1.11 (0.99, 1.23)	0.068	-	-
C16:2-OH	85/163	1.19 (1.03, 1.38)	**0.017**	1.18 (1.01, 1.37)	**0.037**
C16:3-OH/C14:3-DC	82/155	1.28 (0.98, 1.66)	0.067	-	-
C16-OH	85/163	1.02 (0.97, 1.07)	0.40	-	-
C18	85/163	1.0 (1.0, 1.01)	0.36	-	-
C18:1	85/163	1.01 (1.001, 1.01)	**0.027**	1.01 (1.001, 1.01)	**0.018**
C18:1-OH/C16:1-DC	85/163	1.08 (0.98, 1.19)	0.12	-	-
C18:2	85/163	1.01 (1.0, 1.02)	**0.047**	1.01 (1.001, 1.02)	**0.028**
C18:3	83/161	1.10 (1.01, 1.20)	**0.036**	1.12 (1.02, 1.23)	**0.019**
C18-OH/C16-DC	85/163	1.03 (0.98, 1.07)	0.28	-	-
C20	85/163	1.02 (0.90, 1.16)	0.76	-	-
C20:1	85/163	1.06 (0.97, 1.15)	0.23	-	-
C20:1-OH/C18:1-DC	85/163	1.05 (0.99, 1.11)	0.10	-	-
C20:2	85/163	1.07 (0.94, 1.23)	0.31	-	-
C20:2-OH/C18:2-DC	85/162	1.09 (0.94, 1.27)	0.26	-	-
C20:3	85/163	1.08 (0.99, 1.18)	0.070	-	-
C20:3-OH/C18:3-DC	79/153	1.04 (0.80, 1.36)	0.77	-	-
C20:4	85/163	1.10 (1.007, 1.19)	**0.033**	1.10 (1.01, 1.20)	**0.038**
C22:1	85/163	1.13 (0.98, 1.29)	0.092	-	-
C22:2	79/154	1.11 (0.89, 1.39)	0.35	-	-
C22:4	83/160	1.23 (0.89, 1.69)	0.21	-	-
C22:5	83/161	1.28 (1.004, 1.63)	**0.046**	1.31 (1.01, 1.71)	**0.043**

*adjusted for diabetes mellitus, female, BMI, CV rf>2.
